# Dr. M. J. McElhaney

**Published:** 1908-02

**Authors:** 


Dr. M. J. McElhaney, of Greenville, Pa., died at his home January 6, 1908, of pneumonia as a sequel of influensa, aged 69 years. He graduated from the medical department, University of Buffalo with the class of 1870.

